# Therapeutic Strategies Targeting Anti‐CD47 Therapies in Glioblastoma Multiforme: Lead or Dead End?

**DOI:** 10.1111/jcmm.70889

**Published:** 2025-11-06

**Authors:** Anca Buliman, Marius P. Iordache, Mirela‐Gabriela Irina Protosevici, Cristiana Tanase

**Affiliations:** ^1^ Faculty of Medicine Titu Maiorescu University Bucharest Romania; ^2^ Neurology Department Ilfov County Clinical Emergency Hospital Bucharest Romania; ^3^ “Victor Babeș” National Institute of Pathology Bucharest Romania; ^4^ “Nicolae Cajal” Medical Institute – “Titu Maiorescu” University Bucharest Romania

## Abstract

Glioblastoma multiforme (GBM) remains the most aggressive primary brain tumour in adults, characterised by marked cellular heterogeneity, stem‐like subpopulations, and profound resistance to standard therapies. The failure of conventional approaches underscores the need for novel immunotherapeutic strategies that can effectively overcome tumour immune evasion. Multiple therapeutic strategies have been explored to disrupt CD47 signalling in GBM, including monoclonal antibodies, soluble SIRPα fusion proteins, recombinant or peptide fragments derived from TSP‐1, dual CD47/CD36 inhibitors, and antisense oligonucleotides. Overall, anti‐CD47 therapy represents a promising but incomplete strategy in GBM treatment. Its success will likely depend on multimodal approaches integrating surgical, cytotoxic and immune‐modulatory interventions that also target glioma stem‐like populations. Rational combinations that address both immune suppression and tumour heterogeneity could transform CD47 inhibition from a transient lead into a viable therapeutic path for this intractable malignancy.

## 1 Background

Glioblastoma multiforme (GBM) represents the most aggressive primary brain malignancy in adults, with an annual incidence of 3.26 cases per 100,000 [1]. Despite the current standard of care, comprising maximal safe surgical resection followed by radiotherapy and chemotherapy, prognosis remains poor, with a median overall survival of roughly 15 months and a 5‐year survival rate of less than 5% [2]. The pronounced intra‐tumoral heterogeneity and cellular plasticity that characterise GBM are driven by a complex interplay of genetic alterations, epigenetic dysregulation, [3] and differences in the cellular origin of tumour subpopulations. While standard therapies effectively target rapidly dividing tumour cells, a resistant subset known as glioma stem cells (GSCs), characterised by their self‐renewal capacity, multilineage differentiation potential, and ability to initiate and sustain tumour growth, is thought to underlie therapeutic resistance and disease recurrence [2, 4].

## 2 Cellular and Molecular Mechanisms

**FIGURE 1:**
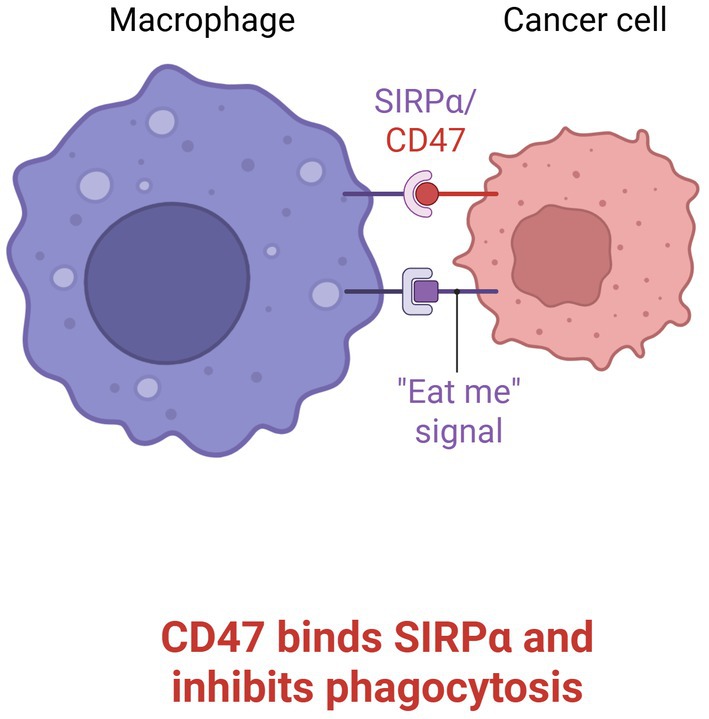
Interaction between CD47 and SIRPα (created with bioRender).

Macrophages, microglia, and other myeloid‐derived cells constitute approximately 30%–50% of the cellular composition in GBM. While lineage‐tracing studies suggest that most of these cells are derived from peripheral monocytes, brain‐resident microglia are also thought to play a role in GBM progression. Tumour cells present surface ‘eat me’ signals (Figure 1) such as calreticulin, high mobility group box 1 protein (HMGB1), and phosphatidylserine (PS), which are recognised by macrophages, leading to phagocytosis. To evade immune clearance, GBM cells increasingly express anti‐phagocytic signals, most notably cluster of differentiation 47 (CD47), a 50‐kilodalton transmembrane protein member of the immunoglobulin superfamily, expressed on most normal and malignant cells [3]. It binds multiple ligands, thereby regulating key cellular processes such as proliferation, apoptosis, migration, phagocytosis, and T cell activation.

The interaction of CD47 with SIRPα, predominantly expressed on myeloid cells, activates immunoreceptor tyrosine‐based inhibitory motifs (ITIMs) within the cytoplasmic domain of SIRPα, leading to recruitment of the phosphatases SHP1 and SHP2 (Src homology region 2 domain‐containing phosphatases 1 and 2). This cascade inhibits myosin accumulation at the phagocytic synapse and transmits a ‘don't eat me’ signal, effectively suppressing phagocytosis (Figure 1). In the GBM microenvironment, this CD47–SIRPα axis promotes immune evasion by supporting the polarisation of tumour‐associated macrophages (TAMs) toward an immunosuppressive M2 phenotype. Conversely, CD47 blockade has been shown to shift TAMs toward the pro‐inflammatory M1 phenotype, enhancing anti‐tumour immunity and facilitating phagocytic clearance [3].

The interaction of CD47 with TSP‐1 (Thrombospondin‐1) contributes to tumour progression by inhibiting angiogenesis. This effect is partly mediated by suppression of nitric oxide (NO) signalling, a known promoter of neovascularization. In addition to its vascular effects, the TSP‐1/CD47 pathway also exhibits anti‐inflammatory activity. This is achieved through the inhibition of key transcription factors, including nuclear factor kappa‐light‐chain‐enhancer of activated B cells (NF‐κB) and activator protein 1 (AP‐1), both of which play central roles in the regulation of inflammatory gene expression [3].

On another path, epidermal growth factor receptor (EGFR) activation enhances CD47 expression by promoting the binding of cellular sarcoma kinase (c‐Src) and phosphorylation at tyrosine 288 (Y288). This post‐translational modification prevents recognition by the E3 ubiquitin ligase tripartite motif‐containing protein 21 (TRIM21), thereby inhibiting CD47 degradation and stabilising its expression. Mutating Y288 reduces CD47 levels, enhances phagocytosis, and limits tumour growth, while preventing CD47 ubiquitination has the opposite effect. Therapeutically, CD47 blockade enhances the efficacy of EGFR‐targeted treatments, and CD47 expression in GBM correlates with both EGFR and c‐Src activity, highlighting a cooperative immune evasion mechanism through the EGFR–c‐Src–TRIM21–CD47 axis [5]. Additional regulation involves tumour necrosis factor receptor‐associated factor 2 (TRAF2), which binds to the cytoplasmic tail of CD47 and promotes its ubiquitination. This disrupts CD47 interaction with microtubule‐associated protein 1A/1B‐light chain 3 (LC3), preventing autophagic degradation and further contributing to immune evasion by suppressing phagocytosis [6].

Through these ligand‐mediated interactions, CD47 plays a vital role in maintaining immune homeostasis and regulating cellular behaviour under physiological conditions [7].

**FIGURE 2:**
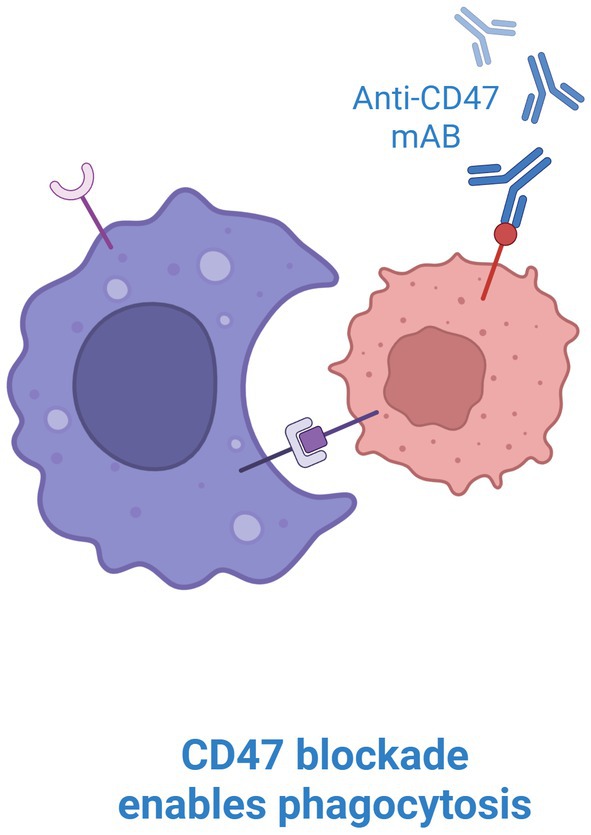
Monoclonal antibody blocking CD47 (created with bioRender).

Despite promising preclinical outcomes with monoclonal antibodies (Figure 2) such as magrolimab (Hu5F9‐G4), clinical trials of CD47 blockade in solid tumours have shown limited efficacy. One likely limitation is the high expression of CD47 on non‐tumour cells, including erythrocytes, which may reduce therapeutic specificity [1].

TIGIT (T cell immune receptor with Ig and ITIM domains) is a co‐inhibitory receptor expressed on multiple immune cell subsets, where it modulates immune responses through interactions with distinct ligands. These interactions suppress natural killer (NK) cell–mediated cytotoxicity and impair dendritic cell–mediated antigen presentation, ultimately attenuating T cell–driven antitumor immunity [8].

## 3 Therapeutic Strategies

Recent advances highlighted strategies that aim to disrupt the expression or activation of CD47 through diverse molecular approaches.

One promising strategy involves disrupting the CD47–SIRPα signalling axis using soluble recombinant fusion proteins encoding the N‐terminal domain of SIRPα, which have shown efficacy in hematologic and solid tumours. A high‐affinity variant, Velcro‐CD47 (N3612), enhances macrophage‐mediated phagocytosis and selectively targets monocytes, with further evaluation underway.

Other therapeutic approaches include recombinant TSP‐1 fragments such as 3TSR and 3TSR/TRAIL fusion proteins, which modulate CD47, with similar clinical endpoints including prolonged survival and manageable side effects. Non‐peptide small molecules mimicking TSP‐1's type 3 repeat region, particularly the FGF‐2 binding site, have also demonstrated antiangiogenic effects in vitro and ex vivo, and computational models continue to guide the development of optimised derivatives for in vivo evaluation.

Epidermal growth factor receptor (EGFR) is frequently dysregulated in glioblastoma (GBM); however, monotherapy with EGFR inhibitors has demonstrated only limited clinical efficacy [1].

Another major avenue involves monoclonal antibodies (mAbs) directed against CD47, some of which are currently undergoing clinical trials. In parallel, peptide‐based approaches have emerged, including TSP‐1‐derived peptides such as 4N1/4N1K, 7N3, and PKHB1, which incorporate the VVM motif critical for CD47 binding, as well as CD47‐derived peptides such as TAX2, which selectively block the TSP‐1/CD47 interaction. However, the therapeutic utility of peptides like 4N1K remains controversial. Although repeated administration of 4N1K has been shown to reduce tumour growth and Ki‐67 expression in preclinical models, subsequent research indicated that these effects might be due to nonspecific protein binding rather than specific CD47 engagement, necessitating cautious interpretation of results. TAX2 has been shown to induce tumour necrosis, reduce vascularization, impair invasion under hypoxic conditions, and enhance anti‐tumour efficacy when combined with bevacizumab.

Targeting both CD47 and CD36 simultaneously has led to the development of dual inhibitors like VT1021, a cyclic pentapeptide currently being evaluated for safety in solid tumours, including glioblastoma.

Finally, gene silencing approaches such as antisense oligonucleotides aimed at reducing CD47 mRNA translation are under investigation in preclinical models, offering yet another potential mechanism to downregulate CD47 and restore immune surveillance [3].

## 4 Risks & Outcomes

CD47 is consistently overexpressed in GBM, particularly in glioblastoma stem‐like cells, where it is associated with higher tumour grade, therapy resistance, and poor clinical outcomes. CD47‐positive GBM cells display features of cancer stem cells, reinforcing their role in tumour aggressiveness and immune escape [8].

Knowing that TRAF2 inhibits CD47 autophagic degradation, it has been shown that combined therapy with a CD47 antibody and rapamycin significantly increased phagocytosis in comparison to monotherapy [6].

Elevated co‐expression of CD47 and TIGIT has been correlated with adverse clinical outcomes, suggesting that patients exhibiting high levels of both markers may be suitable candidates for combined immunotherapeutic strategies targeting CD47 and TIGIT.

A primary challenge associated with CD47‐targeted therapies is the potential for hematologic toxicity, including anaemia, thrombocytopenia, and leukopenia, due to the widespread expression of CD47 on normal erythrocytes and platelets. Although preclinical studies in murine models have indicated that CD47 blockade is generally well tolerated and does not produce overt signs of systemic toxicity, a critical objective remains the optimization of therapeutic strategies to minimise off‐target effects while maintaining robust antitumor efficacy [8].

Functional assays revealed that siRNA‐mediated silencing of CD47 reduced invasive capacity in vitro, while its overexpression enhanced invasion. These findings indicate that CD47 may serve as a potential biomarker of poor prognosis in GBM [9, 10].

For instance, the macrophage checkpoint inhibitor 5F9, when administered in combination with rituximab, demonstrated encouraging therapeutic activity in patients with both aggressive and indolent forms of lymphoma, without evidence of clinically significant toxicity [11].

A notable concordance was observed between the effects of different protein tyrosine kinase inhibitors on the expression of signal transduction proteins and corresponding cellular behaviours, such as adhesion and proliferation, as assessed through real‐time experimental analysis [12].

## 5 Conclusion

While targeting anti‐CD 47 therapies represents a following lead, it is not a ‘silver bullet’, since effective glioblastoma (GBM) therapy necessitates a multifaceted strategy that combines surgery, radiotherapy, cytotoxic chemotherapy (e.g., temozolomide) to induce tumour cell death, combined with immunomodulatory interventions aimed at inhibiting M2 macrophage polarisation, promoting M1 phenotypes, and disrupting immune evasion mechanisms such as phagocytosis checkpoints [13].

Reevaluation of EGFR‐targeted therapies may also be warranted, as EGFR signalling not only drives tumorigenesis but also enhances CD47 protein stability, further facilitating immune resistance [1].

Moreover, complete tumour eradication will require the successful targeting of glioma stem‐like cells (GSCs), which are critical for recurrence and therapeutic resistance. The heightened reliance of GSCs on CD47 expression presents a compelling opportunity for the development of CD47‐directed therapies to eliminate this resilient subpopulation.

## Author Contributions

**Anca Buliman:** conceptualization, investigation, writing – original draft, methodology, software, formal analysis, resources. **Marius P. Iordache:** conceptualization, investigation, writing – original draft, methodology, visualization, formal analysis, resources. **Mirela‐Gabriela Irina Protosevici:** conceptualization, investigation, writing – original draft, methodology, validation, visualization, formal analysis, resources, supervision. **Cristiana Tanase:** conceptualization, methodology, validation, visualization, formal analysis, project administration, data curation, supervision, resources.

## Conflicts of Interest

The authors declare no conflicts of interest.

## Data Availability

This communication does not report any new data. All information presented is derived from previously published literature and publicly accessible sources, as cited in the reference list. No new datasets were generated or analyzed during the preparation of this manuscript.

## References

[1] A. Afzal , Z. Afzal , S. Bizink , et al., “Phagocytosis Checkpoints in Glioblastoma: CD47 and Beyond,” Current Issues in Molecular Biology 46 (2024): 7795–7811, 10.3390/cimb46080462. 39194679 PMC11352848

[2] E. Li , H. Qiao , J. Sun , et al., “Cuproptosis‐Related Gene Expression Is Associated With Immune Infiltration and CD47/CD24 Expression in Glioblastoma, and a Risk Score Based on These Genes Can Predict the Survival and Prognosis of Patients,” Frontiers in Oncology 13 (2023): 1011476, 10.3389/fonc.2023.1011476. 37546426 PMC10399623

[3] C. Tanase , A. M. Enciu , E. Codrici , et al., “Fatty Acids, CD36, Thrombospondin‐1, and CD47 in Glioblastoma: Together and/or Separately?,” International Journal of Molecular Sciences 23 (2022): 604, 10.3390/ijms23020604. 35054787 PMC8776193

[4] T. Sun , B. Liu , Y. Cao , Y. Li , L. Cai , and W. Yang , “AMPK‐Mediated CD47 H3K4 Methylation Promotes Phagocytosis Evasion of Glioma Stem Cells Post‐Radiotherapy,” Cancer Letters 583 (2024): 216605, 10.1016/j.canlet.2023.216605. 38218171

[5] L. Du , Z. Su , S. Wang , et al., “EGFR‐Induced and c‐Src‐Mediated CD47 Phosphorylation Inhibits TRIM21‐Dependent Polyubiquitylation and Degradation of CD47 to Promote Tumor Immune Evasion,” Advancement of Science 10 (2023): e2206380, 10.1002/advs.202206380. PMC10520678 37541303

[6] Q. Gou , B. Yan , Y. Duan , et al., “Ubiquitination of CD47 Regulates Innate Anti‐Tumor Immune Response,” Advancement of Science 12 (2025): e2412205, 10.1002/advs.202412205. PMC11792004 39665172

[7] H. Wu , J. Liu , Z. Wang , W. Yuan , and L. Chen , “Prospects of Antibodies Targeting CD47 or CD24 in the Treatment of Glioblastoma,” CNS Neuroscience & Therapeutics 27 (2021): 1105–1117, 10.1111/cns.13714. 34363319 PMC8446212

[8] J. Hu , Q. Xiao , M. Dong , D. Guo , X. Wu , and B. Wang , “Glioblastoma Immunotherapy Targeting the Innate Immune Checkpoint CD47‐SIRPα Axis,” Frontiers in Immunology 11 (2020): 593219, 10.3389/fimmu.2020.593219. 33329583 PMC7728717

[9] L. Ma , Y. Shi , C. Li , et al., “MGMT Unmethylation and High Levels of CD47 and TIGIT Indicate a Poor Prognosis in Adult Diffuse Gliomas,” Frontiers in Immunology 15 (2024): 1323307, 10.3389/fimmu.2024.1323307. 38404571 PMC10884119

[10] X. Liu , X. Wu , Y. Wang , et al., “CD47 Promotes Human Glioblastoma Invasion Through Activation of the PI3K/Akt Pathway,” Oncology Research 27 (2019): 415–422, 10.3727/096504018x15155538502359. 29321087 PMC7848455

[11] R. Advani , I. Flinn , L. Popplewell , et al., “CD47 Blockade by Hu5F9‐G4 and Rituximab in Non‐Hodgkin's Lymphoma,” New England Journal of Medicine 379 (2018): 1711–1721, 10.1056/nejmoa1807315. 30380386 PMC8058634

[12] C. Tanase , M. L. Cruceru , A.‐M. Enciu , et al., “Signal Transduction Molecule Patterns Indicating Potential Glioblastoma Therapy Approaches,” Oncotargets and Therapy 6 (2013): 1737–1749, 10.2147/ott.s52365. 24348050 PMC3848931

[13] A. C. Tan , D. M. Ashley , G. Y. López , M. Malinzak , H. S. Friedman , and M. Khasraw , “Management of Glioblastoma: State of the Art and Future Directions,” CA: A Cancer Journal for Clinicians 70 (2020): 299–312, 10.3322/caac.21613. 32478924

